# Some New Remedies in Use

**Published:** 1911-03-25

**Authors:** N. Stuart Twist

**Affiliations:** Senior Casualty House Surgeon, Hull Royal Infirmary.


### The General Practitioner's Column.

[Contributions to this Column are invited, and if accepted, will be paid for.]

### SOME NEW REMEDIES IN USE.

By N. STUART TWIST, M.B., Ch.B Leeds, Senior Casualty House Surgeon, Hull Royal Infirmary.

Recently aluminium acetate has been much recommended as a dressing for burns. I have used it in many cases, both of slight and considerable superficial extent, with great success. The dressing is very pleasant to the patient and eases pain. It is used as a 1-per-cent. solution in water. A piece of gauze is soaked in this and applied to the burnt surface. It is kept continually moist with the lotion, and the dressing is reapplied twice daily, and the surface cleaned. With this treatment burns and scalds heal remarkably quickly.

A large "matchbox" burn of the palm of the hand will be quite healed within five or six days. This is much quicker than is the case with the oily or the dry powdery dressings commonly used for burns. Picric acid stains all it comes in contact with, but aluminium acetate is not open to this objection. It is also very cheap, being even cheaper than picric acid.

Burns of considerable superficial extent do well when dressed with it, but they stand no better chance of recovery from the use of it, if the surface is a very large one. It is important that the dressing be kept continually moist. To do this it is not necessary to take it off, and it is, in fact, probably better that this should not be done too frequently. The same solution of aluminium acetate has been recommended as a dressing after circumcisions. It is certainly a good dressing, but is not so superior to others used as it is in the case of burns and scalds.

Lately tincture of iodine has been much recommended for cleaning wounds, and for preparing surfaces for operation. The ordinary pharmaceutical preparation of 2 per cent. strength is generally used. In preparing for an operation, the surface is first thoroughly cleaned with some non-aqueous solution, such as methylated ether (or methylated spirits, which is cheaper); the iodine solution is then applied with a swab, and the part is ready for the incision. Suppuration is extremely rare when this method is used. If only from the point of its simplicity this method of preparation has much to recommend it. For ordinary wounds, the part is dried and all bleeding stopped. For the iodine to exert its full power it must not come in contact with water, so that methylated ether must be used to get rid of any dirt or grease, and then the tr. iodi may be applied. As a matter of fact, the majority of wounds have been washed before they are brought for treatment, but if they be dried and the tr. iodi applied, they do quite well and practically never go wrong. The tr. iodi should be allowed to come in contact with the wound as well as the surrounding parts. In wounds into which dirt has been ground in, it is well to use a stronger solution of iodine—about 7 per cent., being a mixture of the tincture and liniment, is a useful strength—although probably these would do quite well with the ordinary tr. iodi. The tincture of iodine is used for practically all wounds in the casualty room here, and no ill results have been noticed from its use. I have used the lin. iodi, which is 11 per cent. strength, occasionally, and it has not been followed by sloughing even in badly crushed and torn wounds. An objection to the stronger solutions, such as the liniment, is that they make the eyes "water" to such an extent that it is difficult to operate; this is especially the case when iodine solution is made up with methylated spirit.

Carbon-dioxide snow is another recent method of treatment which is being largely used at present. It is by far the simplest method of destroying nævi. All that one requires is a cylinder of $CO_2$, a towel, a glass tube, and a glass rod to use as a ramrod within the tube. The towel is rolled up to leave a bore of the width of an ordinary pint-bottle cork. The cork is put into one end of the rolled-up towel and the neck of the $CO_2$ cylinder into the other, and the gas allowed to escape with considerable force into the towel. After a few seconds the towel may be unrolled, and it will contain the snow required. This is put into the glass tube and rammed down, until a hard pencil of it is formed in the tube. This is pushed out, and can be sharpened with a knife into any shape or size. The snow must not be touched with the bare fingers, but with a pair of ordinary washleather or kid gloves on. This pencil of snow is applied directly to the nævus, and is allowed to remain in contact with it for 20 to 40 seconds. When it is removed, the surface will look white, but this will disappear in a few minutes and the part will regain its former appearance. In a few days there will be slight superficial ulceration, and some boric ointment may then be applied. Within a fortnight the nævus, or the part of the nævus treated, will look like the normal skin. If only a part of a large nævus has been treated, another part may be treated in the same way a fortnight later, and so on till the whole nævus has been destroyed. Surfaces up to the size of a penny may be treated at one sitting, but it is better not to take such large surfaces at first. No scarring appears to follow this "snow" treatment. There is slight pain, but no more, if even as much, as with the other methods of removing nævi. The $CO_2$ treatment can be carried out with ease by the practitioner.

Another new, or comparatively new, remedy is an adrenalin ointment for hæmorrhoids. It is made up of adrenalin solution (1 in 1,000) ♏ iv. ad ℥ lanolin (or other basis). This relieves not only the bleeding, but the feeling of fulness in the rectum and the other symptoms of this condition. In all cases of "piles" it appears to give relief, and it is a great improvement upon the old gallæ c. opio ointment.

